# Assessing the Food Quality Using Carbon Nanomaterial Based Electrodes by Voltammetric Techniques

**DOI:** 10.3390/bios12121173

**Published:** 2022-12-15

**Authors:** Shashanka Rajendrachari, Nagaraj Basavegowda, Vinayak M Adimule, Baris Avar, Prathap Somu, Saravana Kumar R. M., Kwang-Hyun Baek

**Affiliations:** 1 Department of Metallurgical and Materials Engineering, Bartin University, 74100 Bartin, Turkey; 2 Department of Biotechnology, Yeungnam University, Gyeongsan 38541, Republic of Korea; 3 Angadi Institute of Technology and Management (AITM), Savagaon Road, Belagavi 5800321, Karnataka, India; 4 Department of Metallurgical and Materials Engineering, Zonguldak Bülent Ecevit University, 67100 Zonguldak, Turkey; 5 Department of Biotechnology, Saveetha Institute of Medical and Technical Sciences (SIMATS), Saveetha School of Engineering, Chennai 602105, Tamil Nadu, India

**Keywords:** cyclic voltammetry, differential pulse voltammetry, electrochemical sensors, food safety, graphene

## Abstract

The world is facing a global financial loss and health effects due to food quality adulteration and contamination, which are seriously affecting human health. Synthetic colors, flavors, and preservatives are added to make food more attractive to consumers. Therefore, food safety has become one of the fundamental needs of mankind. Due to the importance of food safety, the world is in great need of developing desirable and accurate methods for determining the quality of food. In recent years, the electrochemical methods have become more popular, due to their simplicity, ease in handling, economics, and specificity in determining food safety. Common food contaminants, such as pesticides, additives, and animal drug residues, cause foods that are most vulnerable to contamination to undergo evaluation frequently. The present review article discusses the electrochemical detection of the above food contaminants using different carbon nanomaterials, such as carbon nanotubes (CNTs), graphene, ordered mesoporous carbon (OMC), carbon dots, boron doped diamond (BDD), and fullerenes. The voltammetric methods, such as cyclic voltammetry (CV) and differential pulse voltammetry (DPV), have been proven to be potential methods for determining food contaminants. The use of carbon-based electrodes has the added advantage of electrochemically sensing the food contaminants due to their excellent sensitivity, specificity, large surface area, high porosity, antifouling, and biocompatibility.

## 1. Introduction

For the last few decades, the world has witnessed an increased number of cases of foodborne illnesses because of the adulteration of different toxic chemicals in foods. Food quality assessment has become one of the fundamental routines to determine the quality of food. Consuming quality food keeps us healthy and fit. The necessary nutrients required by our bodies are supplied by the food we consume every day. The quality of any food mainly depends on appearance, nutritional level, texture, ethical, and sustainable production. Many food companies use toxic chemicals and colors to impart a pleasing appearance to attract consumers. The use of polluted water, pesticides, insecticides, and veterinary drugs interfere with the quality of the food and make it toxic. Therefore, food assessment is one of the most important and immediate needs for human beings to keep us away from many diseases. According to a survey by the World Health Organization, 1 person in every 10 people falls ill because of contaminated food. More than 110 billion US dollars are utilized to cure people who get diseases because of consuming adulterated and contaminated food in undeveloped and few developing countries [1].

Providing food for the population is the biggest challenge, and handling the increase in food demand leads to food adulteration by mixing toxic chemicals to enhance the growth rate by decreasing the time required to grow. This is directly affecting the food chain, creating a pileup of a significant quantity of toxic chemicals, and affecting our health seriously. Therefore, food assessment must be performed more regularly, from growth until consumption. All the food must undergo quality checks during preservation and transportation before consumption. Therefore, there is a huge scope for fabricating economic, simple, robust, and highly sensitive methods for detecting different toxic chemicals in foods [2]. The commonly known food contaminants, such as animal drug residues, pesticides, additives, toxins, and pathogens, can result in severe foodborne diseases. Therefore, assessing the food quality is very important and an immediate sake for the consumers to guarantee the food safety and the quality [3].

Generally, the detection of analytes or molecules present in food materials interferes with the chromophore moieties in the food. Some of the common problems with the use of conventional methods in determining food quality are low sensitivity to redox changes, turbidity, low spectrum resolution, and scattering issues related to the sample [4]. Moreover, the miniaturization and portability of detectors are the biggest disadvantages of conventional methods. Therefore, there is a huge demand for quick, robust, selective, and easy methods, such as voltammetric methods, for determining the food’s quality. They exhibit a higher level of selectivity for the redox reactions, and a faster response. They are very simple, economical, and their portability with unlimited miniaturization [4] has made them an ideal and popular choice for assessing the food quality compared with other analytical methods.

The voltammetric methods, such as cyclic voltammetry (CV) [5,6,7,8], linear sweep voltammetry (LSV) [9,10], differential pulse voltammetry (DPV), and square wave voltammetry (SWV), have been constructively used for food assessment. Among them, CV and DPV have been proven to be the best, simplest, most economical, and most popular voltammetric methods effectively used to determine food quality. The advantage of using CV and DPV methods lies in their ability to detect different analytes at low concentrations in one step. Their efficiency, sensitivity, robustness, stability, and accuracy have made these voltammetric methods one of the most widely used voltammetric methods for detecting toxic chemicals present in food. The efficiency of the assessments mainly depends on the type of electrode used. The electrode material may be nanomaterials, alloys, metal oxide nanomaterials, polymers, ceramic oxides, surfactants, etc. However, carbon-based nanomaterials are more suitable for use either as a whole electrode or as a modifier in graphite paste electrodes [11].

In multiple applications, the working electrode is composed of some vital carbon materials that include ordered mesoporous carbon (OMC), carbon dots (CD), graphene, carbon nanotubes (CNTs), boron-doped diamond (BDD), as well as their nanocomposites. Most of these carbon nanomaterials exhibit a high activation energy barrier, and their transition into another form of carbon is extremely sluggish at room temperature and remains unnoticeable. Therefore, they are considered stable materials under certain standard conditions. The bonding between carbon atoms is covalent and sp^2^ hybridized, and therefore, it is very strong, whereas the bonding between interhexagonal rings is weak due to Van der Waals forces. One of the unique properties of these carbon-based materials is their electrical properties. However, opening up the bandgap of graphene materials is quite a challenging research project, even though many researchers have reported the same. Graphene’s band structure has low on-off ratios, and therefore, opening up the bandgap of graphene without compromising its other properties is quite a challenge. In recent years’ various techniques and methods were reported to improve their bandgap without altering the other properties and they are substrate-induced bandgap opening [12], chemical substitution doping [13,14,15,16], synthesizing their hybrids [17,18,19], quantum confinement [20], molecular doping [21] and charge transfer methods.

Carbon nanomaterials also mentioned that the efficiency of the electrode in assessing food quality is greatly influenced by the morphology of the carbon materials. They exhibit needle, rod, barrel, and spherical shapes of different sizes and depict a varied and wide range of chemical detection [22]. The heterocyclic state of the C-C bonds in the carbon materials has imparted excellent chemical and electronic properties [23]. Their excellent sensing ability is further enhanced by their large surface area, refined structure, interface, dielectric confinement, macroscopic quantum tunneling effects, etc. [24]. On the other hand, noncarbon nanomaterials’ synthesis, their characterization, and their unique properties related to particle size, and surface area are reported in the literature [25,26,27].

The present review focuses on the discussion of different properties of various carbon nanomaterials and their electrochemical sensing applications in determining a wide range of food chemicals. The recent advancement in developing carbon-based nanomaterials for electrochemical sensor applications to assess food safety is also discussed in detail, along with the importance of such techniques. Our concern is that the world must develop stable, highly accurate and sensitive, and robust voltammetric methods and modified electrodes to detect the food.

## 2. Different Types of Carbon-Based Nanocomposite to Assess the Food Quality

**Figure 1:**
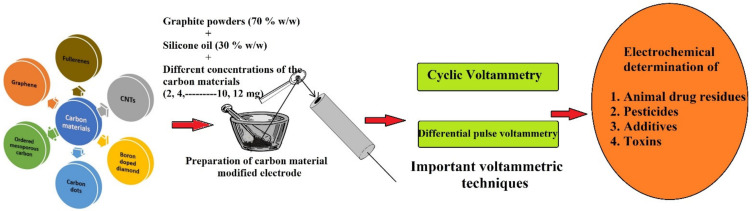
Types of carbon materials and the overview of the article.

Carbon materials, such as CNT, graphene, carbon dots, OMC, and BDD, are generally used as composites to determine food quality. These carbon nanomaterials are most commonly used in electrode materials to assess food quality. These carbon composites exhibit unparalleled characteristics, as evidenced by their differences in surface area and energy, shape and size, thermal and electrical conductivities, surface modifications, etc. Due to these excellent and wide range of characteristics, carbon materials are one of the potentials and are widely used electrode materials for determining food quality. Types of carbon materials and the overview of the article are shown in Figure 1.

### 2.1. Carbon Nanotube (CNT)-Based Nanocomposite for Assessing the Food Quality

S. Ijima, a Japanese scientist, discovered carbon nanotubes (CNTs) in 1991 [28]. Carbon nanotubes are cylindrical tube molecules rolled into a single sheet of graphene. The CNTs can be found as single-walled (SWCNT) with an approximate diameter of 1 nm and sometimes as multiwalled (MWCNT), whose diameter ranges more than 100 nm, whereas their length measures several micrometers to millimeters. They exhibit excellent porosity, a high surface area, and easy surface modification, and some of them act as conductors (armchair-shaped nanotubes) and semiconductors (zigzag-shaped nanotubes) as well.

MWCNTs exhibit good conductivity all the time, and their conductivity can be compared to that of metals [29]. On the other hand, the conductivity of SWCNTs mainly depends on their chiral vector; due to this, they can show electrical conductivity similar to that of metals, and sometimes behave similar to semiconductors or nonconductors. For instance, a small change in the pitch of the helicity can transform the conductivity of CNT from a conductor to a large-gap semiconductor. These unique electrical properties have made the CNTs one of the best, and the most popular choices for fabricating electrodes to assess food quality [30]. The key factors of electrodes, such as their efficiency, repeatability, and sensitivity, depend on multiple features, such as surface modification, the diameter and length of the electrode, and the number of layers present in CNTs. Due to the wide range of excellent properties demonstrated by CNTs; they are the future of electrochemical sensors. They also exhibit significant electrochemical reactivity for a large number of biomolecules and possess the ability to increase electron transfer processes [31]. Therefore, CNTs are excellent carbon materials and can be potentially used either completely as electrodes or in small amounts as modifiers in graphite powder to determine food chemicals [32]. The CNT-based electrochemical sensors are highly sensitive, robust, and show remarkable surface conductivity. The CNTs are sometimes added to various metal nanoparticles that help in the formation of additional sites for the electrocatalytic process, increase sensitivity, and lower the detection limits of the electrodes. Recent electrochemical research is progressively marching towards integrating the conventional biological concepts with digitalization by using instruments (electrochemical sensors) to establish simple, handheld systems that completely depend on specific electrochemical reactions of bioactive compounds and result in electrical, thermal, or optical signals to detect them easily.

Rahemi et al. fabricated MWCNT glassy carbon electrode (GCE) modified by β-cyclodextrin and a polyaniline film to detect the chlorophenoxy herbicide MCPA [33]. This herbicide readily gets absorbed by the leaves and roots of plants; consuming it results in irritation of the skin, serious damage to the eyes, and it also causes drooling, twitching, low blood pressure, unconsciousness, jerking, and spasms. They reported that the fabricated electrode showed excellent electrocatalytic oxidation of the herbicide MCPA with significant sensitivity, repeatability, and stability. They also reported the possible use of the fabricated electrode to detect the presence of MCPA herbicide in real samples, such as natural waters. The use of CV for the present investigation is more advantageous than the sophistically established high-performance liquid chromatography technique. The CV method does not require the previous step’s extraction, cleaning, or derivatization in the range of 10–100 µmol L^−1^. They also reported the obtained detection limit of 0.99 µmol L^−1^ in water [33].

**Figure 2:**
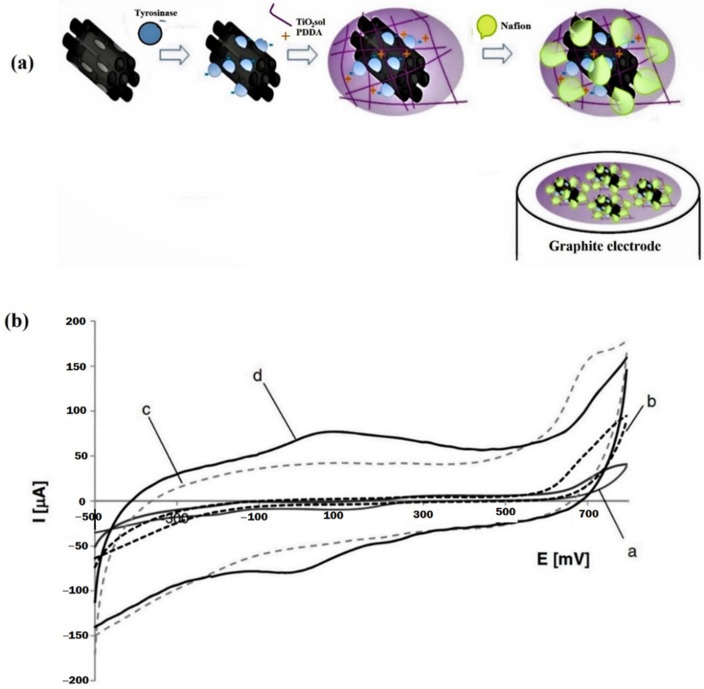
(**a**) Schematic representation of an multi walled carbon nanotube (MWCNT)modified graphite electrode for tyrosine detection, (**b**) Cyclic voltammograms collected at 0.48 mM tyramine solution at **a**: Tyrosinase/TiO_2_, **b**: Tyrosinase/TiO_2_/polycationic polymer/Nafion, **c**: Tyrosinase/TiO_2_/MWCNT/Nafion, and **d**: Tyrosinase/TiO_2_/MWCNT/polycationic polymer/Nafion biosensors. Reprinted (adapted)with permission from Ref. [36]. Copyright 2016, Springer Nature.

Tyrosine produces tyramine during the decarboxylation reaction, which is generally present in chocolate, wine, banana, fish, beer, etc. [34]. Excess consumption of foods containing tyramine can lead to tachycardia, vomiting, strong migraine headaches, rash, hypertonia, palpitation, flushing, rash, etc. [35]. There are reports on the electrochemical determination of tyramine using a MWCNT-modified graphite electrode by cyclic voltammetry [36]. A schematic representation of the MWCNT-modified graphite electrode for tyrosine detection is shown in Figure 2a.

The fabricated sensor electrode demonstrated greater sensitivity and repeatability. Kochana et al. [36] claimed that their fabricated electrode displayed high sensitivity, specificity, and selectivity for the determination of tyramine in the samples of food products. Figure 2b depicts the cyclic voltammograms at 0.48 mM tyramine solution at different modified MWCNT electrodes.

Yan et al. developed an acetylcholinesterase sensor based on the assembly of MWCNTs for the detection of organophosphate pesticides [37]. These pesticides are synthetic chemicals generally used to poison insects and mammals. Intake of this pesticide can directly damage important neurotransmitter enzymes called acetylcholinesterase. Due to the direct damage of the enzyme acetylcholinesterase by organophosphate pesticides, dichlorvos has been applied by the authors as a model compound; the inhibition of dichlorvos was directly proportional to acetylcholinesterase concentration enzyme. They also reported the apparent Michalis–Menten constant (Km) of 0.28 mM for the enzymatic reaction. They claimed that the fabricated electrochemical sensors were simple, robust, highly sensitive, and with utmost stability [37]. Adimule et al. [38,39] reported highly sensitive rare-earth-doped nanomaterials possessing dielectric, EGFET, optical, and photoluminescence properties.

Gonzalez-Fuentes et al. constructed a screen-printed carboxylated SWCNT electrode to determine acrylamide [40]. Acrylamide is a chemical compound used as the precursor to produce polyacrylamide in the polymer industry. It is generally used to treat drinking and residual water; it is also used to process pulp to produce paper and as an additive in cosmetics [41]. Intake of acrylamide can cause neurotoxicity, neuropathy, fertility disorder, and carcinogenesis [40]. The authors have reported that the electroreduction signal of acrylamide is proportional to its concentration at low levels of less than 300 µM. The sensitivity of the electrode was explained in terms of adsorption of acrylamide on the screen-printed carboxylated SSWCNT electrode followed by the electrochemical response of Ferro and ferri-cyanides [40]. The author used this electrode for the determination of acrylamide in fried potatoes using both CV and HPLC methods and found that both methods showed similar results. Yin et al. [42] prepared a sunset yellow dye electrochemical sensor by self-polymerizing the dopamine on the surface of the MWCNTs. The sunset yellow was orange colored azo dye used as a food colorant obtained from petroleum derivatives. If the consumption exceeds 4 mg/kg, it can lead to food intolerance and serious forms of attention deficit hyperactivity disorder (ADHD) in children (but no scientific evidence is available). The authors reported that the fabricated sensor exhibited good selectivity and sensitivity towards the electrochemical detection of sunset yellow dye compared to other reported electrodes. Generally, the enhanced electrocatalytic behavior of CNTs is due to their high specific surface area, highest electrical conductivity, reduced overpotential, and increased surface energy [43,44]. Govindasamy et al. [45] prepared the chloramphenicol (CAP) sensor by modifying the surface of MWCNT with molybdenum disulfide nanosheet (MoS_2_). The chloramphenicol drug causes suspected carcinogenicity and plastic anemia. Therefore, authors studied their electrocatalytic properties at low concentrations. They claimed that the fabricated electrode demonstrated excellent sensitivity even in the presence of large interfering species in milk, powdered milk, and honey samples [45].

As we know, hydrogen peroxide (H_2_O_2_) is produced during various biological and enzyme-catalyzed reactions. The determination of H_2_O_2_ is very important due to its adverse effect on environmental protection, medical diagnostics, and the food industry. Therefore, Tkac and Ruzgas developed an SWCNT electrode for the easy and sensitive detection of H_2_O_2_ [46]. The sensitivity of the electrode was further improved when they dipped the electrode in chitosan solution. Therefore, they reported that the fabricated electrode is significantly dependent on the type of dispersing agent used. Chitosan-dipped electrodes showed higher sensitivity than organic solvents and Nafion-dipped electrodes [46].

**Figure 3:**
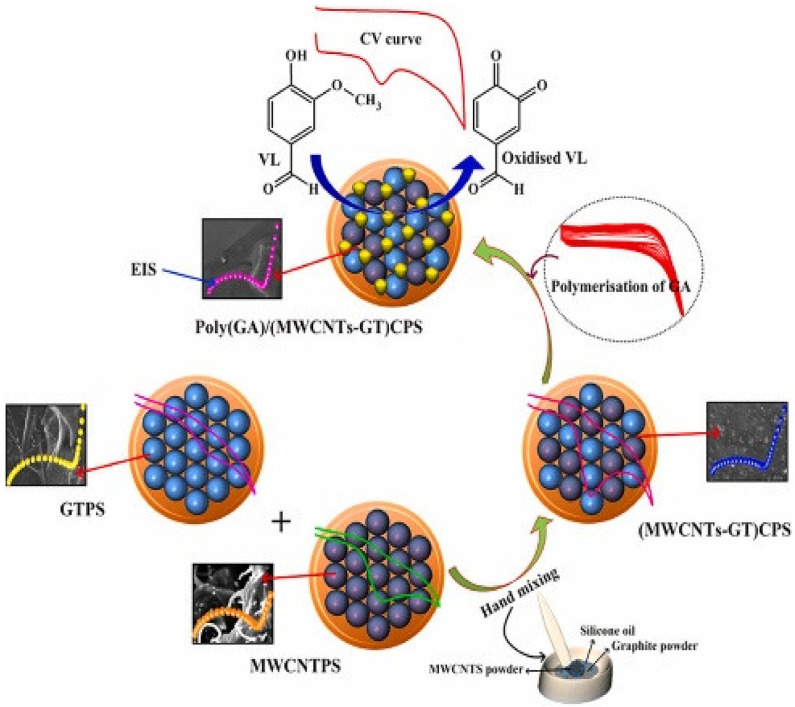
Graphical representation of the whole experiment. Reprinted (adapted) with permission from Ref. [47]. Copyright 2021, Springer Nature.

Hareesha et al. fabricated a fast-responding poly(glutamic acid) functionalized MWCNTs and graphite composite paste electrochemical sensor to determine the vanillin in food samples [47]. Figure 3 depicts the graphical representation of the whole experiment.

They reported the electrochemical oxidation kinetic studies of vanillin at various pHs, scan rates, temperatures, and accumulation times. The modified sensor showed a linear dynamic range of 0.50–18.0 µM for the electrochemical oxidation of vanillin and a low detection limit of 0.0199 µM, respectively. They concluded that the fabricated sensor yielded an adequate recovery for vanillin in different food samples. Further, they claim that the modified sensor is very economical, and exhibits excellent reliability, reproducibility, and repeatability in oxidizing the vanillin in food samples.

### 2.2. Graphene and Related Nanocomposites to Determine the Food Quality

Canadian theoretical physicist P. R. Wallace first discovered graphene in the year 1947, and A. Geim and K. Novoselov were further investigated [48,49,50]. Graphene is considered a super material because of its dynamic mechanical rigidity and thermal stability, but apart from that, it also exhibits unique electrical properties. This extremely unique electrical property of graphene has attracted most electrochemists and researchers to carry out their research on it. The electrical properties depicted by graphene are significantly different from the electrical properties exhibited by other carbon materials, such as CNTs, fullerenes, graphite, etc. [51,52]. The unique physical, chemical, mechanical, and thermal properties of graphene, combined with its excellent light transparency of 97.7%, make it a potential material for the construction of electrodes. Generally, the charge carrier mobility of graphene is found to be approximately 15,000–20,000 cm^2^ V s^−1^ at room temperature. All these properties have made graphene a potential electrode candidate for assessing food quality. Bolotin has reported that the electron’s mobility in graphene layers is at least 100 times greater than that of silicon [53].

Nowadays, graphene is one of the most applied sensor materials in food safety assessment because of its high reactive sites, specific surface area, and charge carrier mobility [54]. The two important types of graphene, reduced graphene oxides (rGO) and graphene oxides (GO), are well reported by Plachá et al. [55] and exhibit slightly different properties. GO is more economical than graphene and requires a very short time for synthesis. Hummer’s process is one of the commonly used methods of preparing GO which uses potassium permanganate and concentrated H_2_SO_4_ as oxidizing agents [56]. GO is acidic because of its hydrophilic nature. The GO sheets can be reused even after dissolving in water. One of the important characteristics of GO is the possibility of converting GO to graphene by different thermal reduction processes [57]. On the other hand, rGO can be synthesized by reducing the GO by laser radiation, electrochemical, or thermal reduction techniques. Preparation of single-layered graphene in solvents is always a tough job and needs extra precautions and experience. Various properties of graphene materials were well reported by Qureshi et al. [58].

The surface of graphene oxide can be modified with carboxyl and carbonyl groups by linking the carbon atoms present in GO with either hydroxy or epoxy groups [59]. Thus, the electrical conductivity increases, followed by the enhanced surface area of the GO. Therefore, GO can be employed as a modifier in the electrode or as a whole electrode to detect different toxic chemicals and food pathogens in foods [59].

The trace of phoxim’s presence in food was detected using an electrode developed by Wu et al. using poly(3-methyl thiophene)/nitrogen-doped graphene material for the construction of the electrode [60]. Phoxim is a type of pesticide that comes under organophosphate pesticides. As we discussed earlier, these types of synthetic pesticides are used to kill insects and mammals and seriously affect the acetylcholinesterase neurotransmitter enzyme. The authors have optimized the cyclic voltammetric conditions and reported that the current varied linearly over two linear ranges (0.02–0.2 µM and 0.2–2.0 µM) concerning the concentration of phoxim. They obtained a low detection limit of 6.4 nM [60].

**Figure 4:**
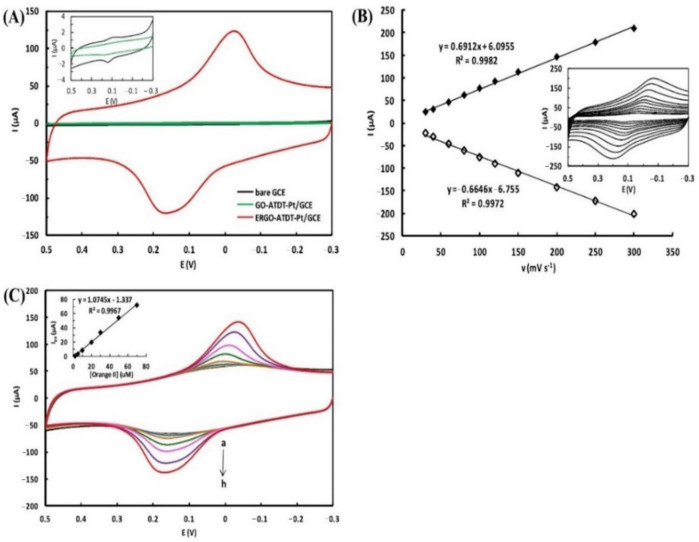
(**A**) The cyclic voltammograms of bare glassy carbon electrode (GCE), 5-amino-1,3,4-thiadiazole-2-thiol-Pt/glassy carbon electrode (ATDT-Pt/GCE), and electrochemically reduced graphene oxide-5-amino-1,3,4-thiadiazole-2-thiol-Pt/glassy carbon electrode (ERGO-ATDT-Pt/GCE) in 50 µM at a scan rate of 100 mV/s; (**B**) the plot of redox peak current vs. scan rate in presence at 30–300 mV/s; (**C**) CV at different concentrations of the orange II dye (0, 2, 5, 10, 20, 30, 50, and 70 µM) Reprinted (adapted) with permission from Ref. [61]. Copyright 2015, Elsevier.

Yun et al. fabricated electrochemically rGO [ERGO] grafted with 5-amino-1,3,4-thiadiazole-2-thiol-Pt [ATDT-Pt] nanoparticles for GCE to detect orange II [61] dye. Orange II is a type of azo dye mostly used in organic LEDs, inks, textiles, hair dyes, shoe polishes, and foodstuffs [61]. It is a carcinogenic dye and decreases the count of red blood cells. Figure 4 shows the CV of bare GCE and modified electrodes during the determination of orange II azo dye.

Yun et al. [61] have detected orange II in 0.1 M acetate buffer solution of pH 4.5 with significant reversible redox peaks. The authors have reported a wide linear range of 1 × 10^−8^–6 × 10^−7^ M and a low detection limit of 3.4 × 10^−10^ M (s/n = 3) for orange II dye detection. The fabricated electrochemical sensor exhibited good sensitivity, robustness, and selectivity for the real samples with significant recovery.

**Figure 5:**
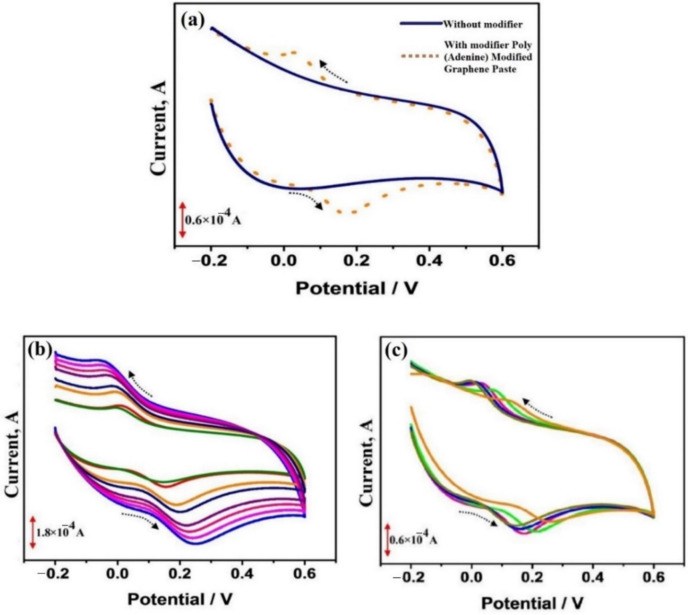
CV curve of catechol using a fabricated electrode at (**a**) 0.1 M PBS (pH 7) at the scan rate of 0.1 V/s. (**b**) Different scan rates from 0.1 to 0.3 V/s. (**c**) Different pH (6.5–8.0) with the sweep rate of 100 mV/s. Reprinted (adapted) with permission from Ref. [62]. Copyright 2020, Bentham Science.

Manjunath et al. reported the catechol determination present in the food by fabricating the voltammetry sensor using poly (adenine) modified graphene [62]. Catechol is one of the toxic organic compounds that can be produced synthetically and used as a precursor to pesticides, flavors, and fragrances [62]. Excessive usage of catechol causes depression in the central nervous system and can raise blood pressure, sometimes it absorbs through the skin, and causes an illness resembling that induced by phenol, except the convulsions are more pronounced. During the catechol determination with the above voltammetry sensor, there was a linear increase in the oxidation peak current of catechol with its concentration in the range of 2 × 10^−6^–8 × 10^−6^ M and 1 × 10^−5^–1.5 × 10^−4^ M with a 2.4 × 10^−7^ M detection limit. The authors used a real water sample to determine the catechol using the developed sensor. Figure 5 depicts the CV curve of catechol using fabricated electrodes at different scan rates and pH.

Shi et al. developed a graphene and thionine nanocomposite electrode to detect fumonisin B1 [63]. Fumonisin B1 is a cardiotoxic chemical generally found in Fusarium verticillioides cultures and naturally contaminated foods [64]. The intake of fumonisin B1 can cause equine leukoencephalomalacia and pulmonary edema in pigs. The surface of the graphene sheet was loaded with a huge number of thionine molecules to enhance its electrical conductivity, surface area, and thus increase the electrochemical signal of the electrode. They reported that the resultant decreased current is proportional to the concentration of fumonisin. They claimed that the fabricated electrochemical sensor exhibits greater sensitivity and selectivity.

**Figure 6:**
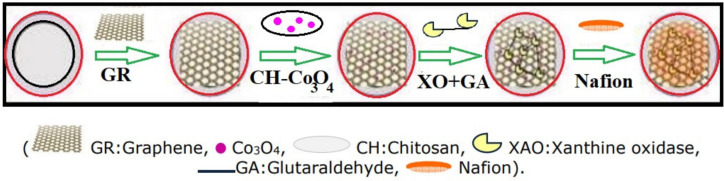
Fabrication process of the modified electrode. Reprinted (adapted) with permission from Ref. [65]. Copyright 2017, Turkish Chemical Society.

Dalkıran et al. constructed a Xanthine electrochemical sensor modified with graphene/cobalt oxide nanoparticles/chitosan composite to determine the freshness of fish [65]. Figure 6 shows the graphical representation of the fabrication of the graphene-modified electrode. Excessive intake of xanthine can cause mild diuresis, anxiety, nervousness, tremors, headaches, and dizziness. The authors have optimized the electrochemical experimental conditions, and they detected xanthine in the concentration range of 5.0 × 10^−4^ –8.0 × 10^−2^ mM with a detection limit of 2.0 × 10^−4^ mM. They reported increased enzymatic affinity for the immobilized enzyme due to the low Michaelis-Menten constant value of 0.17 mM. However, the fabricated sensor showed quite a short time of 10 seconds, a high sensitivity of 6.58 μA/mM, and excellent reproducibility of RSD = 1.2%. The authors used real fish meat to study the xanthine assay.

Ma and Chen [66] fabricated a diethylstilboestrol (DES) sensor using a graphene doped gold nanoparticle-modified electrode. The DES is a veterinary medicine used for the treatment of estrogen-deficiency disorders and is also used as a growth stimulant in animals [67]. The consumption of even the smallest residual traces of DES in meat can cause carcinogenic disorders in humans [66]. The authors reported that the fabricated electrode displayed high sensitivity and specificity in determining the DES even in the presence of interfering ions, such as estradiol, estriol, estrone, and folic acid.

**Figure 7:**
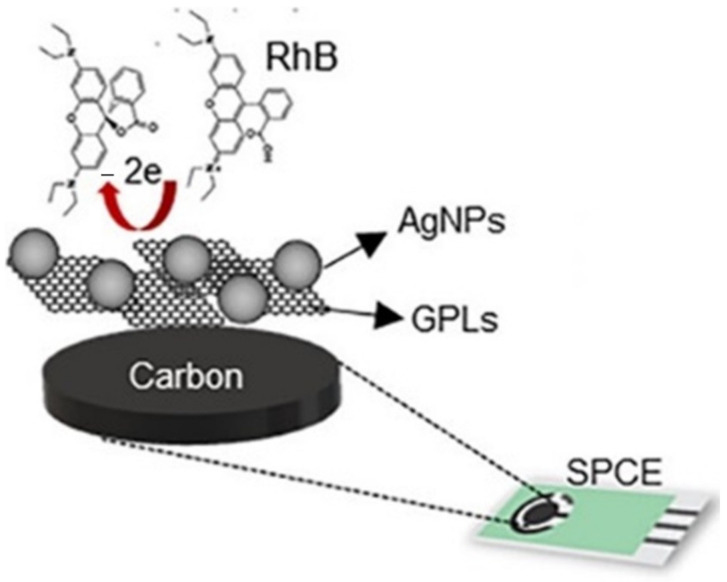
Schematic representation of the silver nanoparticle/graphene nanoplatelets modified screen-printed carbon electrodes. Reprinted (adapted) with permission from Ref. [68]. Copyright 2021, American Chemical Society.

Kartika et al. constructed silver nanoparticle/graphene nanoplatelets on modified screen-printed carbon electrodes for determining rhodamine B (RhB) dye in food products [68]. A schematic representation of the silver NPs/graphene nanoplatelets modified screen-printed carbon electrodes is shown in Figure 7.

RhB dye is used as a food additive and as a colorant in most foods due to its attractive color and low cost. However, it is considered a group III carcinogenic agent [68]. The fabricated electrochemical sensor proved to be highly sensitive to determine RhB with a linear range of 2−100 μM, and with a limit of detection of 1.94 μM. The sensor also exhibited a selective detection of RhB in the presence of interfering compounds, such as sucrose and monosodium glutamate, present in the food samples.

### 2.3. Carbon Dots (CDs) and Their Nanocomposites as Sensors for Assessing the Food Quality

Carbon dots (CDs) are the new grades of the carbon family and have attracted many researchers in the past few years. They are considered quasi-zero-dimensional carbon materials consisting of small carbon atoms with a size of less than 10 nm. They exhibit low toxicity, excellent quantum yield, good photoluminescence, extremely refined size, eco-friendliness, are less expensive, and are easily synthesized. One of the remarkable features of CDs is that their physicochemical properties can be easily regulated using surface passivation and functionalization [69]; thereby allowing the application of CDs in the fabrication of electrocatalysis, chemical, biosensing, and optoelectronic devices. Xu et al. in 2004, accidentally obtained carbon nanoparticles with fluorescence during the purification of SWCNTs [70]. There are mainly three types of CDs available and they are carbonized polymer dots, graphene quantum dots, and carbon quantum dots. This classification is based on their formation mechanisms, micro-/nanostructures, and properties [70].

**Figure 8:**
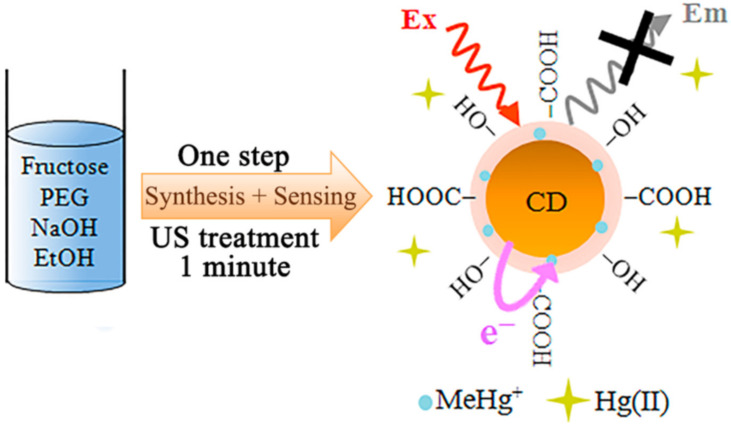
Graphical representation of preparing fluorescent carbon dots on a modified electrode to determine methylmercury. Reprinted (adapted) with permission from [72]. Copyright 2014, Elsevier.

Hou et al. constructed a graphene quantum dots/gold NPs modified GCE for the effective determination of malachite green dyes [71]. These dyes are mainly used as colorants in industry and as antimicrobial agents due to their low cost and availability. However, malachite green is a highly toxic dye, and its excessive intake can cause serious health issues, such as carcinogenesis, mutagenesis, chromosomal fractures, teratogenicity, and respiratory toxicity. Using the fabricated graphene quantum dot electrode, the authors have obtained a pair of quasi-reversible adsorption-controlled redox peaks, respectively, at 0.502 V (Epa) and 0.446 V (Epc) using a 0.05 mol L^−1^ H_2_SO_4_ solution [71]. The fabricated electrode was used for the detection of malachite green in fish samples, and the authors reported that the obtained results are satisfactory, reproducible, and stable with a 96.25–98.00% recovery rate. Costas-Mora et al. [72] electrochemically determined the methylmercury using carbon dot nanoprobe. Methylmercury is a very toxic neurotoxin discharged into the human body after consuming fish and affects the brain and nervous systems of particularly pregnant women and infants [73]. A graphical representation of fabricating the fluorescent carbon dot-modified electrode is shown in Figure 8. Authors claimed to fabricate a highly sensitive and robust methylmercury electrochemical sensor, which can detect the analytes in less than 1 minute even at very low concentrations with the detection limit of 5.9 nM.

Dong et al. [74] developed the amantadine electrochemical sensor using carbon dots. Amantadine is basically not a veterinary drug but was used largely in the poultry industry before it was recently banned in many countries. This antiviral drug can cause nausea and dizziness in humans if there is an excess amount of intake. The fabricated electrode was successful in detecting even the traces of amantadine residues in chicken, with a limit of detection of 0.02 ng mL^−1^. Xiang et al. fabricated graphene quantum dots electrodes to detect hepatitis B virus (HBV) DNA [75]. HBV is a very harmful human pathogen that can infect easily and cause liver inflammation, cirrhosis, and liver cancer. According to the authors, the fabricated electrochemical sensor exhibits high sensitivity with a detection limit of 1 nM, and a linear detection range from 10 to 500 nM. However, they reported that the developed sensor could be a potential candidate for detecting other probe DNA, due to the strong interaction between single-stranded DNA and graphene quantum dots [75].

**Figure 9:**
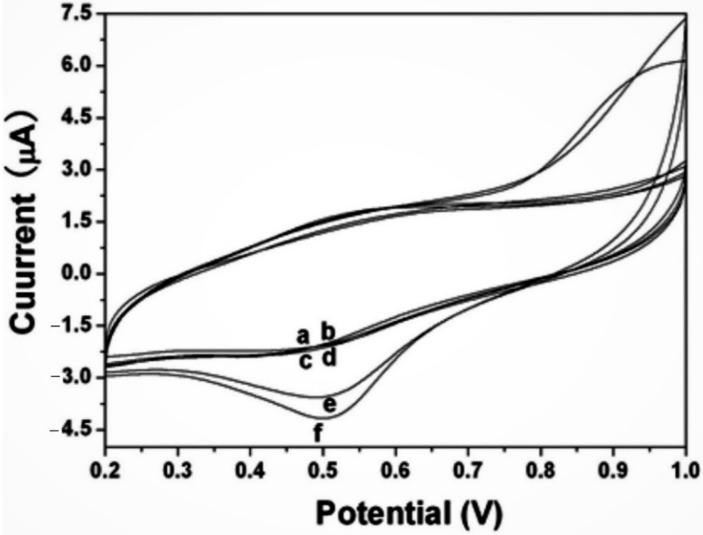
CV curves of GCE (**a**,**b**), carbon dots/GCE (**c**,**d**) and carbob dots@Au/GCE (**e**,**f**) in the absence (**a**,**c**,**e**) and presence (**b**,**d**,**f**) of 0.2 mg/L ractopamine in a PBS (pH 7.0) solution. Reprinted (adapted) with permission from [76]. Copyright 2020, ESG.

Song et al. developed carbon dots that reduced gold NPs for the determination of ractopamine in pork meat [76]. It is an animal feed additive used to increase leanness and food conversion efficiency in farmed animals, but it can cause down syndrome and severe cardiovascular stress in humans after consuming meat containing ractopamine. Figure 9 depicts the CV curves of GCE, carbon dots, and Au-modified GCE to determine ractopamine in a PBS (pH 7.0) solution. The authors have studied the kinetics of redox reactions between the fabricated sensor and the ractopamine. The authors reported that their electrochemical sensor has shown greater sensitivity, good reproducibility, and stability during the determination of ractopamine.

### 2.4. Ordered Mesoporous Carbon (OMC)-Based Nanocomposites for Food Assessment

The porous carbons are the new addition to the family of carbon materials, showing greater porosity with maximum surface area and energy [49]. Due to their excellent chemical inertness, electrical conductivity, high mechanical strength, and ordered and regular structure, this new grade of carbon material is quite famous among researchers and therefore uses electrode materials [77]. The classification, characteristics, and applications of OMC were reported in detail by Shashanka in their previous publication [78]. Libbrecht et al. [79] reported the 2D hexagonal OMC structure in their previous article.

Kochana et al. fabricated mesoporous carbon electrochemical sensors to detect tyramine in food products [36]. Their consumption can cause strong migraine headaches, vomiting, tachycardia, rash, hypertonia, palpitation, flushing, rash, etc. Authors have reported that the developed mesoporous carbon electrochemical sensor electrocatalytically oxidized tyramine with a linear range from 6 to 130 μM, and a detection limit of 1.5μM, respectively. They also reported the high biological affinity of the fabricated sensor against tyramine due to the low Michaelis–Menten constant [80] (66 μM). The efficiency of the sensor was evaluated in food products as well and showed excellent sensitivity, repeatability, and limits in detection. Yang et al. [81] fabricated a ractopamine sensor using an OMC-modified electrode by the cyclic voltammetric method. The ractopamine is a toxic veterinary drug used as heart tonics, bronchodilators, and tocolytics. Ractopamine accumulates in animal tissue, and its consumption can cause frequent vomiting, muscular tremors, and cardiac palpitations [81]. Authors reported the oxidation mechanism of ractopamine along with its electro-oxidation behavior in the presence of an OMC-modified electrode [81]. Finally, the authors concluded that the present electrode can detect the ractopamine in pork samples with high accuracy, sensitivity, and selectivity. Nanomaterials of rare earth metals are also used for the construction of electrical and electronic devices due to the noticeable changes in their structure, morphology, and reactivity [82,83].

Guo et al. developed an OMC-modified GCE for the detection of melamine [84] in milk products. Melamine is mainly used to produce melamine-formaldehyde resins and is also used as a filler for protein-rich diets in milk powder. Excessive intake of melamine causes kidney stones, traces of blood in urine, high blood pressure, and little to no urine production. The fabricated electrode, in the presence of 0.1 M copper ions converts, non-electroactive melamine into an electroactive Cu-melamine complex [84].

### 2.5. Boron Doped Diamond (BDD)-Based Nanocomposites for Food Quality Assessment

Most carbon materials are highly conductive [85,86,87,88]. However, a natural or pure diamond cannot be used as the material for the construction of an electrode due to its excellent electrical insulation properties. However, the conductivity of diamond materials can be enhanced by p-doping approximately 1018 and 1021 atom cm^−3^ with boron. Boron-doped diamond is proven to have one of the largest potential windows of all electrode materials, with significant conductivity, stability, robustness, low noise, chemical inertness, biocompatibility, resistance to passivation, and comparatively less fouling and background current. Due to a wide range of properties, BDD is considered one of the strongest candidates for electrode materials to determine toxic food chemicals.

Švorc et al. used a miniaturized boron-doped diamond electrode to detect theobromine in chocolate products [89]. The main source of theobromine (a dimethylxanthine alkaloid) is cocoa, chocolate, and other related products. It can cause reduced yields of cattle milk, thymus atrophy in rats, retarded growth, and lethargy in pigs. Under optimized experimental conditions, the linear calibration curve for theobromine was observed in the concentration range of 0.99–54.5 μM with a sensitivity of 0.07 μA/μM. Micheletti et al. [90] carried out voltammetric determination of one of the toxic azo dyes, carmoisine E-122, in food using a cathodically pretreated BDD electrode. Commonly, the synthetic carmoisine E-122 is used in many foods, such as chewing gum, candies, sauces, and beverages, to provide a red to maroon color to the food [90]. However, excessive consumption of carmoisine E-122 has an adverse effect on the renal and hepatic functions and results in hyperactivity in children [91]. The authors have reported that the analyst is linear over the concentration range of 0.059–1.31 μmol L^−1^ with limits of detection of 7.0 for an anodic process. The authors reported that the prepared electrode successfully determined the carmoisine E-122 dye present in both surface water and food samples with maximum sensitivity [90]. Medeiros et al. [92] simultaneously determined different types of synthetic dyes present in food using a single electrode (a cathodically pretreated BDD electrode). Most of the synthetic dyes contain toxic chemicals and can cause cancer, asthma, and many other diseases. Usually, these dyes are added to sweets, sugar candies, beverages, jellybeans, etc., to make them more visually appealing to customers [92,93]. Chuanuwatanakul et al. [94] fabricated a BDD-based electrode to determine chloramphenicol (a veterinary drug) using cyclic voltammetry. This antibiotic drug was very popular in treating and preventing bacterial infections in animals due to its low cost and is mostly used in aquaculture farming. However, it is banned all over the world because it can cause aplastic anemia, agranulocytosis, and many more diseases in humans [95].

### 2.6. Fullerenes and Their Nanocomposites for Food Quality Assessment

Fullerene is also called a buckyball and it is an allotrope of carbon wherein single and double bonds involved in carbon atoms connect to form a fused ring having 5–7 carbon atoms. Fullerenes may be hollow spheres, ellipsoids, or many other sizes and shapes. Generally, there are different types of fullerenes available based on the number of carbon atoms present in the molecule. It is possible to prepare C_70_, C_76_, C_78_, C_84_, C_100_, and C_240_ fullerenes. These new compounds of carbon materials exhibit unique and unexpected properties. The extraordinary electrical conductivity of fullerenes makes it a potential candidate for fabricating electrochemical sensors for determining food quality.

Tajeu et al. developed fullerene/MWCNT/Nafion-modified GCE for the detection of caffeine [96]. Most of the pharmaceutical and food industries use caffeine in tea, coffee, and soft drinks. Excessive use of caffeine causes increased gastric acid secretion and diuresis and can stimulate the central nervous and cardiovascular systems. The fabricated sensor provides an irreversible oxidation peak of caffeine at approximately +1.33 V in HClO_4_. The authors reported that the electron transfer process is diffusion-controlled [96]. The fabricated electrode shows excellent stability even in the presence of interfering compounds. Therefore, the authors claimed that the fabricated sensor is a potential candidate for the detection of caffeine in various foods and drugs. Table 1 depicts the carbon-based nanocomposites as electrochemical sensors for food assessment.

All the tabulated carbon-based nanocomposites are used as potential electrochemical sensors for determining various toxic chemicals present in food products. Toxic chemicals, such as carmoisine E-122, theobromine, chloramphenicol, ractopamine, melamine, methylmercury, organophosphate pesticides, malachite green, norfloxacin [101,102], and many more, are present due to the adulteration of food products, either as a food dye, veterinary drug, preservative, excessive use of pesticides, etc. Most of these studies are still under laboratory development, and this technology has yet to come to the market in the form of a digital instrument.

## 3. The Challenges and the Future of Assessing Food Quality

Most countries have a few organizations that monitor food quality regularly. But, the major problem of food quality analysis is a lack of qualified personnel, unable to meet the huge demand for quality assessment, and the lack of sufficient data on the contamination starting from the farm to the consumption level. Especially, in highly populated countries, local food safety is always a question mark, and food quality organizations may not perform a quality assessment of all the food available. Lack of scientific knowledge to process the food is also a big problem. Therefore, there is a need to create awareness about food safety, the impact of food-borne diseases, the processing of food among people, and the need to develop toxicological data for all the possible types of chemicals present in food, irrespective of their concentrations. More research work must be conducted to improve the efficiency of the available voltammetric techniques and to develop new techniques to assess food quality.

## 4. Conclusions

CV and DPV are the two most common and popular voltammetric methods used for the qualitative analysis of food products. The present review article discusses the latest developments in carbon composite electrochemical sensors for assessing food quality. Their extraordinary electronic properties and the different morphologies of carbon materials make them useful as electrochemical sensors for determining a wide range of toxic chemicals present in food. These types of carbon can be used either as modifiers or as whole electrodes to increase detection efficiency. We know that a huge number of nanomaterials, polymers, and alloys are employed in fabricating electrochemical sensors to detect food quality. However, incorporating carbon nanostructures into the above common electrode materials and chemically building 2D and 3D structures of carbon materials can boost the sensitivity of the electrodes and improve their efficiency. The main advantages of using carbon materials in food sensors are the availability of a high surface area, excellent current response, robustness, selectivity, and multicomponent detection. The main disadvantage of using carbon materials as a whole electrode instead of modifying them with graphite powder and silicone oil is that some of the carbon materials are very expensive. The second disadvantage is the instability of the carbon materials with few of the food chemicals; therefore, the analyte and the modifier must be chosen wisely. Therefore, it is recommended to add the carbon materials to the carbon paste electrodes in low concentrations of 2–10 mg for better results. Currently, many researchers are electrochemically determining the toxic chemicals in food by using theoretical and experimental approaches with greater accuracy in the results. We have considerably reviewed numerous theoretical and practical studies and described the preparation of fabricating carbon-nanomaterial-based electrochemical sensors with possible modifications and application in food safety testing. However, sensitivity and selectivity of food sensing needs may be enhanced with increased stability and reusability at the point of economic feasibility possible by improving fabricating technique, selections of construction materials, the incorporation of additional reaction sites, and surface area to increase significant electrochemical sensing ability toward food assessment.

## Figures and Tables

**Table 1:** Carbon material-based nanocomposites as electrochemical sensors for food assessment.

Type of Carbon Material (Electrode)	Type of Voltammetric Techique Used	Analytes	Linear Range	Detection Limit	Type of Sample Used	Reference
MWCNTs	Cyclic voltammetry	Herbicide MCPA	10–100 μM	0.99 μM	Natural water	[33]
SWCNTs	Cyclic voltammetry	Acrylamide	10–200 μM	0.03 μM	Fried potatoes	[40]
MWCNTs	Cyclic voltammetry	Organophosphates pesticides	2.00 to 10.00 µM	0.68 ± 0.076 µg/L	Food	[37]
MWCNTs	DPV	Malathion	0.1–700 μM	0.1 nM	Food	[97]
rGO	Cyclic voltammetry	Orange II	10–600 nM	0.34 nM	Chili sauce and ketchup	[68]
Graphene	Cyclic voltammetry	Aflatoxin B1	3.2 fM–0.32 pM	1 fM	Spiked food	[98]
Graphene	Cyclic voltammetry	Sudan I	0.075–7.50 μM	40 nM	Ketchup	[99]
Graphene	Cyclic voltammetry	Fumonisins B1	1–10^6^ pg/mL	1 pg/mL	Feed	[63]
Graphene	Cyclic voltammetry	Xanthine	5.0 × 10^−4^ to 8.0 × 10^−2^ mM	2.0 × 10^−4^ mM	Fish	[65]
Graphene	Cyclic voltammetry and DPV	Rhodamine B	2–100 μM	1.94 μM	Food	[68]
CDs	Cyclic voltammetry	H_2_O_2_	10 μM to 7.38 mM	0.15 μM		[100]
Graphene quantum dots	DPV	Malachite green	4.0 × 10^−7^ to 1.0 × 10^−5^ mol L^−1^	1.0 × 10^−7^ mol L^−1^	Food	[71]
Graphene quantum dots	Cyclic voltammetry and DPV	Hepatitis B virus	10 to 500 nM	1 nM		[75]
CDs	Cyclic voltammetry and DPV	Ractopamine	0.01 to 32.5 mg/L	1.2 μg/L	Pork meat	[76]
OMC	Cyclic voltammetry	Tyramine	6 to 130 μM	1.5 μM	Food	[36]
OMC	Cyclic voltammetry and DPV	Ractopamine	0.085 to 8.0 μM	0.06 μM	Pork meat	[81]
OMC	Cyclic voltammetry and DPV	Melamine	1.0 × 10^−8^ to 2.0 × 10^−5^ M	2.0 × 10^−9^ M	Milk products	[84]
BDD	Cyclic voltammetry and DPV	Theobromine	0.99 to 54.5 μM	0.42 μM	Chocolate products	[89]
BDD	Cyclic voltammetry and DPV	Carmoisine E-122	0.059–1.31 μmol	7.0 nmol L^−1^	Surface water and food	[90]
BDD	Cyclic voltammetry	Chloramphenicol	0.1–50 μM	0.03 μM	Milk samples	[94]
Fullerene	Cyclic voltammetry and DPV	Caffeine	10 to 1000 μM	7.29 × 10^−8^ M	Drugs	[96]

## Data Availability

Not applicable.
